# So Many Diagnostic Tests, So Little Time: Review and Preview of *Candida auris* Testing in Clinical and Public Health Laboratories

**DOI:** 10.3389/fmicb.2021.757835

**Published:** 2021-10-07

**Authors:** Emily K. Dennis, Sudha Chaturvedi, Vishnu Chaturvedi

**Affiliations:** ^1^Mycology Laboratory, Wadsworth Center, New York State Department of Health, Albany, NY, United States; ^2^Department of Biomedical Sciences, University at Albany, Albany, NY, United States

**Keywords:** PCR, real-time PCR, MALDI-TOF MS, LAMP, biosensor, laboratory-developed tests, clinical, surveillance, healthcare-associated infections

## Abstract

The recognition of a new yeast, *Candida auris*, in 2009 in East Asia, and its rapid global spread, was a reminder of the threats posed by multidrug-resistant fungal pathogens. *C. auris* had likely remained unrecognized for a long time as accurate tests were not available. The laboratory community responded to the *C. auris* challenge by publishing 35 new or revised diagnostic methods between 2014 and early 2021. The commercial sector also modified existing diagnostic devices. These *C. auris* diagnostic tests run the gamut from traditional culture-based differential and selective media, biochemical assimilations, and rapid protein profiles, as well as culture-independent DNA-based diagnostics. We provide an overview of these developments, especially the tests with validation data that were subsequently adopted for common use. We share a workflow developed in our laboratory to process over 37,000 *C. auris* surveillance samples and 5,000 *C. auris* isolates from the outbreak in the New York metropolitan area. Our preview covers new devices and diagnostic approaches on the horizon based on microfluidics, optics, and nanotechnology. Frontline laboratories need rapid, cheap, stable, and easy-to-implement tests to improve *C. auris* diagnosis, surveillance, patient isolation, admission screening, and environmental control. Among the urgent needs is a lateral flow assay or similar device for presumptive *C. auris* identification. All laboratories will benefit from devices that allow rapid antifungal susceptibility testing, including detection of mutations conferring drug resistance. Hopefully, multiplex test panels are on the horizon for synergy of *C. auris* testing with ongoing surveillance of other healthcare-associated infections. *C. auris* genome analysis has a proven role for outbreak investigations, and diagnostic laboratories need quick access to regional and national genome analysis networks.

## *Candida auris*, a Novel Pathogen

Clinical laboratories made significant progress in the identification of fungal pathogens with the introduction of ribosomal RNA gene sequencing and matrix-assisted laser desorption ionization–time of flight mass spectrometry (MALDI-TOF MS) (White et al., 1990; Qian et al., 2008; Marklein et al., 2009). These DNA- and protein-based approaches enhanced the capacity of many frontline laboratories to recognize new pathogens, which were previously the province of highly specialized centers of excellence (Sullivan et al., 1995; Alcoba-Florez et al., 2005; Sugita et al., 2006; Jensen and Arendrup, 2011; Cendejas-Bueno et al., 2012; Castanheira et al., 2013; Eddouzi et al., 2013; Tsang et al., 2014). This scenario played out perfectly when *Candida auris* was recognized as a new yeast species by Satoh et al. (2009). The authors studied a single yeast isolate from the external ear canal discharge of an elderly patient at a Tokyo metropolitan hospital using biochemical tests and ribosomal RNA gene sequences (ITS and D1-D2) to delineate novel *C. auris* formally (Satoh et al., 2009). Around the same timeframe, Kim et al. (2009) reported 15 isolates of a novel *Candida* species, now confirmed as *C. auris*, from ear canals of chronic otitis media patients in South Korea. These isolates were susceptible to relatively high concentrations of amphotericin B and fluconazole. A subsequent study from South Korea by Lee et al. (2011) established *C. auris* as a causal agent of fatal fungemia with intrinsic and acquired resistance to fluconazole. The two teams highlighted misidentification of *C. auris* by commercial systems. They also emphasized the value of internal transcribed spacer (ITS) sequencing as a confirmatory test (Kim et al., 2009; Lee et al., 2011). Teun Boekhout’s group reclassified *Candida haemulonii* species complex to recognize *C. haemulonii*, *C. haemulonii* var. *vulnera*, *Candida pseudohaemulonii*, *Candida duobushaemulonii*, and *C. auris* (Cendejas-Bueno et al., 2012). Notably, these investigators reported that MALDI-TOF MS was as good as the ITS sequencing for identification purposes (Cendejas-Bueno et al., 2012). Thus, within a short span of 4 years, a rare group of yeast species with intrinsic antifungal resistance was characterized for clinical significance, and the MALDI-TOF MS test was prescribed for rapid laboratory identification.

## *Candida auris* Internal Transcribed Spacer (ITS) and Matrix-Assisted Laser Desorption Ionization–Time of Flight Mass Spectrometry

Subsequent notable developments in the *C. auris* saga involved reports from outside East Asia. Sarma et al. (2013) described two *C. auris* isolates from candidemia cases from a hospital in North India. These isolates were amphotericin B- and fluconazole-resistant. The tally was most likely higher than reported, as only two of 15 isolates tentatively identified as *C. haemulonii* using a commercial yeast kit were sent out for re-identification by ITS sequencing (Sarma et al., 2013). Later, Chowdhury and coworkers described a series of *C. auris* cases from North and South India (Chowdhary et al., 2013, 2014). The authors suggested a clonal population based on amplicon typing and noted nucleotide variability in ITS gene sequences from East Asian *C. auris* isolates (Chowdhary et al., 2013, 2014). The *C. auris* isolates in these series were resistant to fluconazole, voriconazole, caspofungin, and flucytosine (Chowdhary et al., 2014). An additional case of fluconazole-resistant *C. auris* candidemia was reported from Kuwait in 2014, attesting to broader distribution in South Asia and Middle East (Emara et al., 2015). Also, in 2014, four cases of fluconazole-resistant *C. auris* candidemia were described from South Africa, and the investigators reported nucleotide variability in ITS sequences (Magobo et al., 2014). Thus, multiple reports documented widespread misidentification of *C. auris* by available specialized culture media, biochemical test kits, or commercial systems, highlighting the need for ITS sequencing (Chowdhary et al., 2013, 2014; Sarma et al., 2013; Magobo et al., 2014; Won et al., 2014; Emara et al., 2015). Using a more extensive collection of 102 *C. auris* isolates, the Chowdhury group also affirmed an earlier report by Cendejas-Bueno et al. (2012) on the suitability of MALDI-TOF MS as a more facile approach for *C. auris* confirmation (Kathuria et al., 2015; Prakash et al., 2016). In a preliminary study, VITEK MS instrument was found to be efficient in identification of eight of twelve reference strains of *C. auris* (Girard et al., 2016). Ghosh et al. (2015) and Bao et al. (2018) created main spectrum projections (MSP) and an in-house database (CMdb), respectively, to demonstrate it was possible to identify *C. auris* using a commercial Bruker Biotyper MALDI-TOF MS system. Other investigators also generated in-house databases to improve their Bruker MALDI-TOF MS reference library (Ceballos-Garzon et al., 2020). Of note, the US Food and Drug Administration (FDA) approved the BRUKER MALDI Biotyper CA system (April 20, 2018) and the bioMérieux Vitek MS (December 21, 2018) for *C. auris* identification (Zhu et al., 2020).

## *Candida auris* PCR and Loop-Mediated Isothermal Amplification

Polymerase chain reaction (PCR) in many configurations remains the technology of choice for newly described laboratory tests for *C. auris* (Table 1). Among the most straightforward applications, a duplex one-tube ITS-PCR assay was developed to speciate *C. auris* from *C. haemulonii* by the size selection of amplicons (Theill et al., 2018). Another direct PCR application targeted glycosylphosphatidylinositol (GPI) protein-encoding genes to speciate *C. auris* and 18 other *Candida* species by gel visualization of different size amplicons (Ruiz-Gaitan et al., 2018). The Boekhout group described novel tetraplex PCR and 21-Multiplex PCR for *C. auris* and other closely related and other yeast species, and extensive testing with 405 reference strains and 804 clinical strains of yeasts from three different countries (Arastehfar et al., 2018, 2019a,b, 2019c). Prospective and retrospective validations were performed in hospitals that appear not to have *C. auris* (Arastehfar et al., 2018, 2019a). Although targeted for resource-poor settings, direct PCR methods appear not to be widely adopted either due to the potential for contamination, the difficulties of recording minor variations in the gel bands or the unavailability of validation data needed for a laboratory-developed test (LDT) (Bockstahler, 1994; Millar et al., 2002; Hoorfar et al., 2004). A commercial multiplex nucleic acid test panel that includes *C. auris*, received FDA approval on March 18, 2020; we did not find any independent evaluation of this product in peer-reviewed publication (BioFire^®^ Blood Culture Identification 2 (BCID2) Panel, FilmArray^®^ 2.0 or FilmArray^®^ Torch systems, bioMérieux, Inc., Durham, NC, United States).

**TABLE 1:** *Candida auris* culture-independent DNA-based identification and confirmation methods.

**Method**	**Target**	***C. auris* (No. isolates)**	**Closely-Related Yeasts (No. isolates)**	**Other Fungi (No. isolates)**	**Clinical specimen (No.)**	**Sensitivity (%)**	**Specificity (%)**	**LOD (CFU/reaction)**	**References**
**PCR**									
	GPI	139	18	ND (Not Done)	ND	98	100	ND	Ruiz-Gaitan et al., 2018
	ITS2	20	30	ND	ND	100	100	ND	Theill et al., 2018
**Multiplex PCR**									
	26S	138	34	9	Mouse blood and tissue (21)	100	100	ND	Arastehfar et al., 2018
	26S	3	297	ND	ND	100	100	ND	Arastehfar et al., 2019a
	26S	35	1149	47	ND	100	100	ND	Arastehfar et al., 2019c
**Real-time PCR**									
	ITS2	44	92	5	ND	100	100	10	Kordalewska et al., 2017
	ITS2	17	40	31	Swab (365)	89	99	1	Leach et al., 2018
		58			Sponge (258)	100	89	1	Leach et al., 2018
	ITS2	47			Swab (110)	96	92	1	Leach et al., 2019
	ITS2	73			Swab (247)	93.6	97.2	1	Ahmad et al., 2019
	ITS1/2	10	103	13	Simulated sputum, Urine, Wound swabs, and Serum (11)	100	100	1	Lima et al., 2019
	ITS2	32	18	54	Simulated urine, Blood, and Swab (30)	93.3–100	96	4–54	Walchak et al., 2020
	GPI	155	18	ND	Simulated serum (1)	100	100	5	Alvarado et al., 2021
	GPI	8	62	2,123	Stool and water samples (2073)	100	100	13	Ibrahim et al., 2021a
	ITS	4	113	8	ND	100	100	1	Jafarian et al., 2020
LAMP	Pyruvate synthase	20	32	13	ND	100	100	20	Yamamoto et al., 2018
**Commercial**									
Real-time, *Auris*ID		72	40	ND	Swab (113)	96.6	100	1	Mulet Bayona et al., 2021
Real-time, *Auris*ID		29	13	ND	Simulated serum (2)	100	0	1	Sattler et al., 2021
Real-time, Fungiplex Candida Auris RUO		29	13	ND	Simulated serum (2)	100	0	9	Sattler et al., 2021
qPCR, GPS^TM^ MONODOSE dtec		117	25	ND	ND	100	100	5	Martinez-Murcia et al., 2018
qPCR, SYBR green	ITS2	48	ND	55	ND	93	96	4	Sexton et al., 2018b
T2Dx		50	43	ND	ND	89	98	5	Sexton et al., 2018a
GenMark Dx ePlex		49	700	70	Blood (141)	100	100	ND	Zhang et al., 2020
Biosensor	GPI	31	13	ND	Simulated serum (22)	85	100	6	Pla et al., 2021

Yamamoto et al. (2018) developed a loop-mediated isothermal amplification (LAMP)-based approach to detect *C. auris*. LAMP forward/reverse primers targeting *C. auris* the pyruvate: ferredoxin oxidoreductase domain from uncharacterized protein (NCBI CJI97_003625, Gene ID 40028770) were tested in PCR at 56°C for 90 min and the product visualized with a turbidimeter (Yamamoto et al., 2018). The assay had an excellent limit of detection (LOD) (20 copies/reaction) and specificity (100%) with a large panel of *C. auris* and other fungi, positive *C. auris* identification from a clinical swab, and simulated environmental specimens (Yamamoto et al., 2018). Surprisingly, *C. auris* LAMP assay has not seen broader adaptation, perhaps because test kit manufacturers appear to be unenthused about LAMP technology (Cantera et al., 2019).

## *Candida auris* Real-Time PCR

Kordalewska et al. (2017) described a real-time PCR assay for *C. auris* (Table 1). The new assay targeted *Candida* ribosomal genes for specific primers used either for direct PCR and detection of variable amplicon sizes or for real-time PCR and melting curve analysis of the double-strand-specific dye SYBR^®^ Green I (Wittwer et al., 2013). The investigators reported 100% accuracy (100% sensitivity and specificity) for *C. auris* from closely related species based on a proficiency panel of 44. *C. auris* and 97 other yeast isolates (Kordalewska et al., 2017). Further evaluation and modification of the assay by the Perlin laboratory and CDC investigators allowed direct detection of *C. auris* from patient swabs with 93% sensitivity and 96% specificity (Sexton et al., 2018b). This milestone marked the availability of a rapid *C. auris* real-time PCR test for surveillance purposes with the accompanying validation data required of an LDT. Within 3 months of the publication of the new real-time assay, one of us (SC) led a team that developed and validated a TaqMan-based real-time PCR assay targeting the ribosomal ITS2 of *C. auris* (Leach et al., 2018). The validation study comprised 623 surveillance samples, including 365 patient swabs and 258 environmental sponges. We found 49 swabs and 58 sponge samples positive, with 89 and 100% clinical sensitivity vis-a-vis culture-positive results (Leach et al., 2018). The distinguishing features of our assay vis-à-vis earlier publication from Perlin laboratory were the use of TaqMan probe chemistry, higher sensitivity (with an LOD of 1 *C. auris* CFU/PCR), the inclusion of all known clades of *C. auris* as reported by whole-genome sequencing, and direct utilization of the test for detection of *C. auris* from large numbers of surveillance samples (Kordalewska et al., 2017; Leach et al., 2018; Sexton et al., 2018b). We further expanded our manual assay to an automated sample-to-result real-time *C. auris* PCR assay using the BD Max open system (Leach et al., 2019). The new assay, with culture as gold standard, yielded 96% clinical sensitivity, and 94% clinical accuracy with 110 patient surveillance samples (Leach et al., 2019). The new assay appeared promising for broader adaptability and availability of high throughput surveillance testing (Leach et al., 2019). CDC investigators adapted our manual assay to an even higher throughput platform by automating the extraction steps and achieved diagnostic sensitivity and specificity of 93.6 and 97.2%, respectively (Ahmad et al., 2019). Both manual and semi-automated *C. auris* assays developed by our group were adopted by other laboratories, including the CDC Antifungal Resistance Lab Network (personal communications) (Caceres et al., 2019; Malczynski et al., 2020; Pacilli et al., 2020; CDC, 2021a). A few months before submission/publication of our BD Max assay, Lima et al. (2019) described a BD Max assay for *C. auris*. The authors designed primers to target many fungal ribosomal genes and tested a collection of fungi and bacteria and 50 contrived clinical specimens to report 100% clinical sensitivity and specificity (Lima et al., 2019). The authors indicated that their primer-design strategy was superior to other reported assays, and the new assay was widely applicable. However, only 10 *C. auris* isolates and no *C. auris* positive clinical or surveillance samples were tested during the validation steps (Lima et al., 2019). Further information is awaited about the performance and adoption of the assay in other laboratories.

In 2020–2021, several additional laboratories described real-time PCR assays for *C. auris* (Walchak et al., 2020; Alvarado et al., 2021; Ibrahim et al., 2021a; Table 1). Commercial primers and probes, melting curve analysis, and validation with limited *C. auris* isolates and contrived clinical samples allowed ≤100 CFU/reaction sensitivity from blood and urine (Walchak et al., 2020). A similar melting curve approach using primers against GPI-modified protein-encoding genes allowed specific identification and detection of *C. auris* with an LOD of 5 CFU/reaction for isolates, 20 CFU/reaction from spiked blood and serum (Alvarado et al., 2021). A TaqMan-chemistry assay with an ITS2-specific probe achieved LOD 1 CFU/reaction when four *C. auris* isolates and other fungal and bacterial strains were tested (Jafarian et al., 2020). Another study utilizing a GPI-target probe with TaqMan-chemistry and simulated samples achieved an LOD of 13 *C. auris* CFU/qPCR reaction (Ibrahim et al., 2021a). The number of publications suggests real-time PCR is a preferred approach for rapid identification of *C. auris* from clinical, surveillance, and environmental samples. However, in the absence of head-to-head comparisons, it is not clear if SYBR^®^ Green I or TaqMan chemistry, or a particular real-time machine, are preferable for *C. auris* real-time PCR assays. A few studies not involving *C. auris* describe the relative merits of various real-time probes. Moreover, a process was published to compare in-development real-time platforms; we refer the readers to these studies to make an informed choice about primers, probes, and a platform suitable for their laboratory needs (Van Poucke et al., 2012; Polinski et al., 2013; Ahrberg and Neužil, 2015; Cantera et al., 2019).

## *Candida auris* Real-Time PCR Kits

*Auris*ID^®^ is a commercial kit with ready-made reagents for qPCR to identify *C. auris* from fungal culture (OLM Diagnostics, Newcastle upon Tyne, England) (Table 1). A retrospective evaluation with *C. auris* positive swab samples reported 96.6% sensitivity (Mulet Bayona et al., 2021). In a second study, *Auris*ID^®^ detected *C. auris* with an LOD of 1 genome copy/reaction but gave false positives with high DNA amounts of the *C. haemulonii*, *C. duobushaemulonii*, and *C. pseudohaemulonii* (Sattler et al., 2021). Fungiplex^®^ Candida Auris is a real-time, research use only (RUO) PCR assay for the rapid detection of *C. auris* in hospital hygiene applications including ready-made reagents for qPCR (Bruker Daltonics GmbH & Co. KG. Bremen, Germany). An independent evaluation reported the Fungiplex^®^ Candida Auris kit LOD to be 9 copies/reaction, much lower than *Auris*ID^®^ (LOD = one copy/reaction) and 100% specificity for five *C. auris* isolates tested (Sattler et al., 2021). The investigators also tested Fungiplex^®^ Candida Auris in “off-label use” with blood samples spiked with two *C. auris* isolates to obtain ∼45 viable CFU/reaction (Sattler et al., 2021). All three commercial ready-to-use real-time PCR reagent kits do not disclose primer and probe details, which may or may not impact their future use as new *C. auris* clades are discovered. None of these kits have current regulatory approval for routine use in diagnostic laboratories.

## Differential and Selective Media

Differential culture media continue to be widely used in busy clinical laboratories. CHROMagar^TM^ Candida Plus is a new chromogenic differential medium (Table 2). A comparative evaluation with HiCrome *C. auris* MDR selective agar, CandiSelect, CHROMagar^TM^ *Candida*, and Chromatic *Candida* commercial media revealed *C. auris* colonies develop a species-specific coloration, as do closely related pathogenic species *C. pseudohaemulonii* and *Candida vulturna* (de Jong et al., 2021). In a similarly designed laboratory evaluation, CHROMagar^TM^ Candida Plus agar was judged to be an excellent alternative to conventional mycological media for the screening of patients with *C. auris*, as only *Candida diddensiae* yielded a similar coloration (Borman et al., 2021). An earlier evaluation of CHROMagar^TM^ Candida Plus found 100% sensitivity and specificity for *C. auris* when 14 surveillance samples were tested (Mulet Bayona et al., 2020). Even in an off-label modification, CHROMagar Candida was reported to offer differentiation between *C. auris* and *C. haemulonii* complex (Kumar et al., 2017). Thus, CHROMagar Candida Plus agar, and possibly other commercial differential media, hold promise for presumptive identification of *C. auris* when the laboratory confirms the identification with MALDI-TOF MS or another confirmatory test as per the manufacturer’s recommendation. Welsh et al. (2017) described the first selective medium when they reported *C. auris* growth at an elevated temperature (40°C) and salinity (10% wt/vol) in the Sabouraud or yeast nitrogen base broths with dulcitol or mannitol as the carbon source. The high salt medium had an excellent performance in the selective enrichment of *C. auris* cells from patient and environmental surveillance samples (Zhu et al., 2020; Sexton et al., 2021). A modified Selective Auris Medium (SAM) was recently described with YPD agar comprising 12.5% NaCl and 9 mM ferrous sulfate and incubation at 42°C (Das et al., 2021). Another variation of selective medium described by Walsh is termed SCA (specific *C. auris*) medium, incorporating crystal violet to prevent the growth of *Candida tropicalis* (Ibrahim et al., 2021b). Both SAM and SCA are reported to improve the original *C. auris* selective medium, but independent confirmations are not yet available.

**TABLE 2:** *Candida auris* identification by mass spectrometry and other biochemical methods.

**Method**	***C. auris* (No. isolates)**	**Closely-Related Yeasts (No. isolates)**	**Other Yeasts (No. isolates)**	**Clinical specimen (No.)**	**Sensitivity (%)**	**Specificity (%)**	**References**
**Mass spectrometry**							
Bruker	90	12	ND	ND	100	100	Kathuria et al., 2015
Bruker	82	11	ND	ND	100	100	Girard et al., 2016
Bruker, CMdb database	33	62	16	ND	100	100	Bao et al., 2018
MALDI	3	298	ND	ND	100	100	Arastehfar et al., 2019a
MALDI, PXD016387 database	300	8	ND	ND	100	100	Ceballos-Garzon et al., 2020
Autof MS 1000	2	1216	ND	ND	100	100	Yi et al., 2021
Vitek MS	2	1216	ND	ND	100	100	Yi et al., 2021
Biotyper	61	ND	ND	ND	75.4–83.6	ND	Kwon et al., 2019
Vitek MS	61	ND	ND	ND	93.4–96.7	ND	Kwon et al., 2019
**Differential media**							
CHROMagar^TM^ with Pal’s medium	15	13	ND	ND	100	100	Kumar et al., 2017
CHROMagar^TM^ *Candida* Plus	37	58	ND	swab (23)	100	100	Mulet Bayona et al., 2020
CHROMagar^TM^ *Candida* Plus	10	52	ND	ND	ND	98	Borman et al., 2021
CHROMagar^TM^ *Candida* Plus	9	35	ND	ND	90	100	de Jong et al., 2021
**Selective media**							
Selective Auris Medium	133	446	ND	Bactec blood culture broth (40)	100	100	Das et al., 2021
Specific *C. auris* (SCA) Medium	7	128	50	stool (200)	100	100	Ibrahim et al., 2021b

In laboratories without access to ITS sequencing and MALDI-TOF MS, it is convenient to use manual or automated biochemical panels and systems to confirm the identification of yeast isolated from culture. Earlier reports and recent re-evaluations found incomplete or incorrect identifications of *C. auris* by the API ID 32 C system (version 4.0 database), AuxaColor^TM^ 2 (Bio-Rad Laboratories, Marnes-la-Coquette, France), Vitek 2 (bioMérieux, Marcy l’Étoile, France), BD Phoenix (BD Diagnostics, Sparks, MD, United States), and RapID Yeast Plus (Remel, Thermo Fisher Scientific, Lenexa, KS, United States) (Won et al., 2014; Kathuria et al., 2015; Kim et al., 2016; Ruiz Gaitan et al., 2017; Iguchi et al., 2018; Sharp et al., 2018; Snayd et al., 2018; Ambaraghassi et al., 2019; Tan et al., 2019; CDC, 2021b; Du et al., 2021; Table 2). A recent report on API ID 32 C concluded that *C. auris* could be identified if the percentage of positive reactions is registered in the database or calculated manually (Du et al., 2021). It appears these panels and systems need further refinements and evaluation to assess their utility for the identification of rare yeasts, including *C. auris* (Du et al., 2021). It might also be prudent for the end users to promptly install the latest product updates from the manufacturers and review any new information on approvals and evaluations.

## *Candida auris* Antifungal Resistance Testing

Antifungal susceptibility testing (AFST) of *C. auris* is performed with CLSI or EUCAST methods and commercial devices (Chowdhary et al., 2014, 2018; Arendrup et al., 2017; Bidaud et al., 2019; Escandon et al., 2019; Kwon et al., 2019; O’Brien et al., 2020; Zhu et al., 2020; CDC, 2021a). Several new drugs in development were also tested for their efficacy against *C. auris* using standard AFST (Arendrup et al., 2018; Ghannoum et al., 2020). A few studies describe antifungal combination testing for *C. auris* using LDTs (Fakhim et al., 2017; Bidaud et al., 2019; O’Brien et al., 2020). The literature on *C. auris* antifungal testing is voluminous, and discussion of these publications is not possible here. The focus is on the evaluation of established or new laboratory tests. Nevertheless, it is incumbent upon laboratories to perform both identification and antifungal testing for *C. auris* as part of their diagnostic service. The drug-resistance pattern in *C. auris* remains variable. Also, regional patterns have been observed for fluconazole and other triazoles, echinocandins, and amphotericin B (Chowdhary et al., 2018; Escandon et al., 2019). There is emerging evidence about the utility of susceptibility testing in selecting appropriate antifungal drugs in the management of patients (Alatoom et al., 2018; Chen et al., 2018; Dewaele et al., 2020). Lessons learned from *C. auris* outbreak investigations included a vital role for antifungal susceptibility data for surveillance purposes, including monitoring emerging drug resistance patterns in the community (Mulet Bayona et al., 2020; Ostrowsky et al., 2020; Zhu et al., 2020; Tian et al., 2021). AFST test innovations for *C. auris* remain sparse, with just one report describing same-day identification and echinocandin-resistance testing using MALDI-TOF MS (Vatanshenassan et al., 2019). Further confirmations and correlation of the new test approach with standard AFST are not yet available. The phenotype based CLSI, EUCAST, and commercial AFST tests are slow. Ideally, phenotype tests should be preceded by rapid DNA tests for drug-resistant *C. auris*, ensuring timely treatment of infected patients and effective pathogen control measures. In promising developments, Perlin laboratory described a molecular beacon-based platform for detecting *FKS1* (echinocandins) and *ERG11* (azoles) mutations in *C. auris* isolates and patient swab samples (Hou et al., 2019; Kordalewska et al., 2019). The molecular tests for drug resistance are desirable as surrogates for standard AFST tests standalone or multiplexed with rapid ID tests for *C. auris*. Molecular platforms offer flexibility to target new mutations encountered in drug-resistant *C. auris*.

## *Candida auris* Diagnostic Algorithm

**FIGURE 1:**
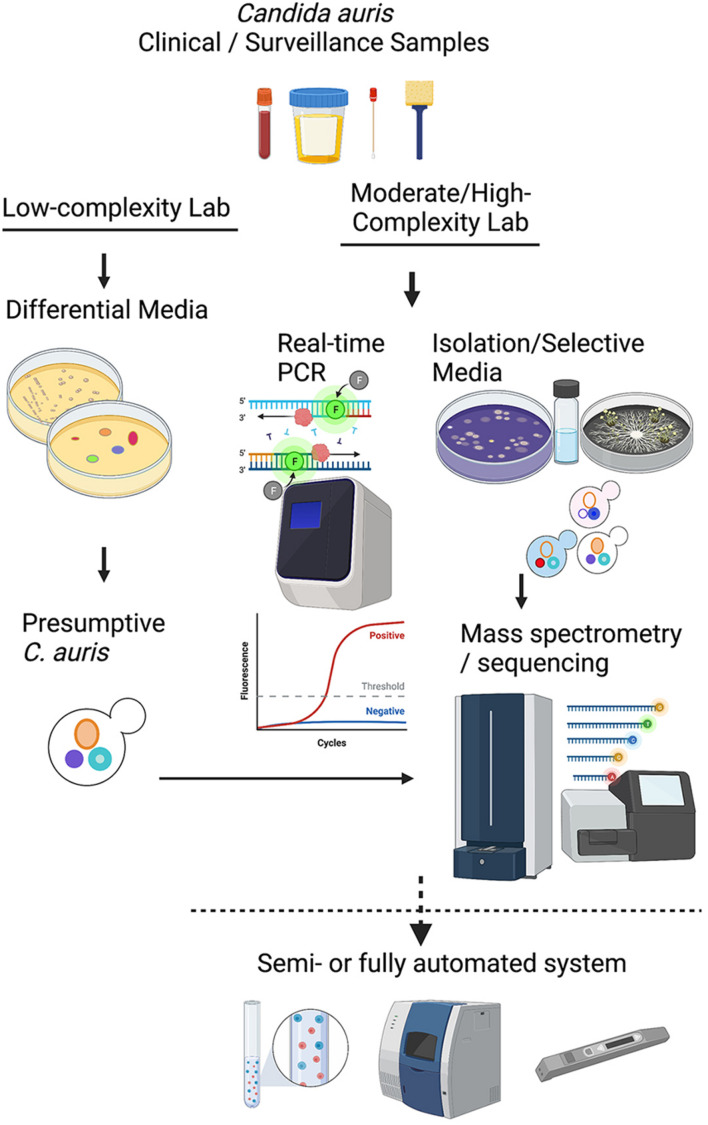
A conceptual illustration of testing method differences between low-complexity and moderate- to high-complexity laboratories.

**FIGURE 2:**
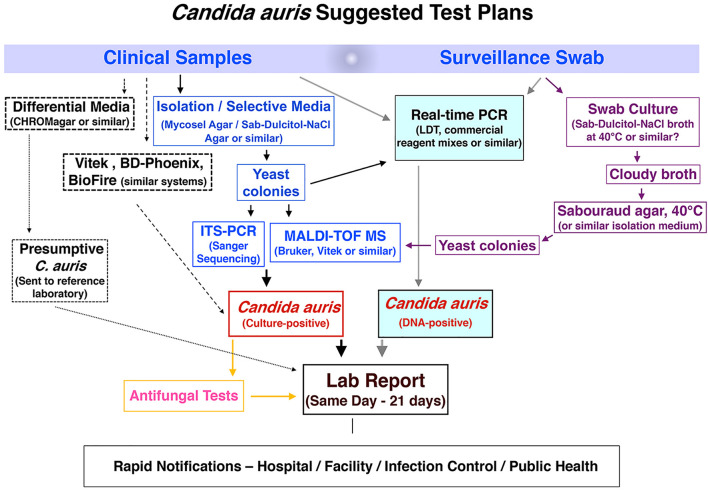
Workflow algorithm for high-volume surveillance and clinical sample testing of *C. auris*. Dotted lines indicate alternate laboratory approaches.

*Candida auris* colonization and infection among hospitalized patients and long-term care residents have become a global problem, with localized outbreaks reported from several countries. Several laboratory modes of operation are possible regarding pathogen isolation, rapid identification, susceptibility testing, and genotyping. Figure 1 provides a conceptual illustration of testing method differences between low-complexity and moderate- to high-complexity laboratories. A significant challenge for specialized diagnostic mycology laboratories concerns supporting *C. auris* surveillance and outbreak investigations. Such a service is available in large bacteriology laboratories but seldom needed for fungal pathogens. Consequently, many clinical recommendations and guidelines were published for laboratories that work with or want to expand their services for *C. auris* (Lockhart et al., 2017; Tsay et al., 2018; Caceres et al., 2019; Kenters et al., 2019). In the United States, the latest versions of the CLSI M-54 document and the Manual of Clinical Microbiology, peer-recommended guidelines for diagnostic microbiology laboratories, do not have *C. auris*-specific guidelines for isolation and identification (Carroll et al., 2019; CLSI, 2021). Early in 2017, we devised an algorithm which has been modified regularly since then to cope with an unprecedented volume of surveillance and clinical samples of *C. auris*. Alternate laboratory approaches are shown with dotted lines. We share the scheme hoping it will help other laboratories to modify their workflows to suit local needs (Figure 2).

## *Candida auris* Diagnostics on the Horizon

Among commercial products likely to be available soon, T2MR (T2 magnetic resonance) is a portable system that detects candidemia by measuring signals from PCR-amplified *Candida* nanoparticle clusters in the blood directly within 3 h (Neely et al., 2013; Mylonakis et al., 2015). In 2018, CDC investigators evaluated a T2Cauris panel (T2 Biosystems, Lexington, MA, United States) with axilla/groin swab samples and reported 89% sensitivity and 98% specificity for detecting *C. auris* (Sexton et al., 2018a; Table 2). The T2Biosystems^®^ website states that T2Cauris is not cleared for diagnostic testing and is available for research use only (RUO). MONODOSE dtec-qPCR *C. auris* is a commercial ready-to-use qPCR kit for pathogen detection (Genetic PCR Solutions^TM^, Alicante, Spain). The manufacturer completed a validation study with academic partners according to UNE-EN ISO/IEC 17025:2005 standard (Martinez-Murcia et al., 2018). The investigators reported MONODOSE dtec-qPCR *C. auris* passed validation in two independent laboratories and is ready to undergo clinical evaluation (Martinez-Murcia et al., 2018). Among new sample-to-answer systems, ePlex BCID-FP is an investigational use only proprietary reagent test panel for 15 *Candida* species used with a proprietary ePlex System (GenMark Diagnostics, Inc., Carlsbad, CA, United States). A multi-laboratory evaluation with 3 *C. auris* isolates and 49 contrived blood samples obtained 100% sensitivity and specificity (Zhang et al., 2020). Another sample-to-answer approach based on an oligonucleotide-functionalized gated nanosystem for *C. auris* achieved 85% sensitivity and 100% specificity in limited testing with 22 blood samples (Pla et al., 2021). Mass spectrometry systems from manufacturers other than Bruker are also available and in use in clinical laboratories. A minimal evaluation with two *C. auris* isolates reported equal efficacy of Vitek MS and Autof MS 1000 systems (Yi et al., 2021). It is safe to say that any MALDI-TOF MS machine will provide identification of *C. auris* provided enough representative isolates encompassing all known clades are included in the database to train the decision algorithm (van Belkum et al., 2017). Limited but promising results are also being published on sample processing and machine learning applications to MALDI platforms, which will benefit *C. auris* diagnostics eventually (Muthu et al., 2018; Weis et al., 2020; De Bruyne et al., 2021).

## *Candida auris* Biosafety, Disinfectants, and Advisories

*Candida auris* poses unique occupational risks for laboratory personnel due to potential exposure to multidrug-resistant organisms. The reported persistence of *C. auris* on inanimate objects requires focused efforts at environmental decontamination. No *C. auris*-specific guidelines are available in the latest edition of BMBL Biosafety in Microbiological and Biomedical Laboratories (DHH, 2020). CDC and many state and local jurisdictions in the United States have issued *C. auris* advisories for healthcare professionals. The guidance information is regularly updated online for easy consultation. The United States EPA’s (Environmental Protection Agency) List P includes 23 disinfectant products with claims about their ability to kill *C. auris* (EPA, 2021). We follow institutional biosafety guidelines for BSL 2 laboratories. Our additional precautions include frequent changes of hand gloves and disposable lab apparel. The laboratory work surfaces are decontaminated pre- and post-procedure with freshly prepared 10% bleach solution, followed by 70% ethanol. We secure fungal cultures in secondary containers for transfer and incubation within the laboratory. Periodic sampling of space and instruments are done by *C. auris* real-time PCR and culture to check for any inadvertent fungal contamination.

## *Candida auris* Test Wish List

The availability of rapid *C. auris* DNA tests onsite remain severely restricted, especially in resource-poor settings. The results are available only after 24–72 h, assuming samples are sent out for reference testing. DNA tests are challenging for many frontline laboratories and long-term care facilities due to a lack of equipment and trained personnel. MALDI-TOF MS assays work best with isolated *C. auris* colonies, but the time required delays diagnosis by 3–7 days on average (Zhu et al., 2020). Frontline laboratories need rapid and facile onsite testing of *C. auris* to inform their efforts at identification, surveillance, patient isolation, admission screening, and environmental control (Durante et al., 2018; Wang et al., 2020). Thus, there is an unmet, urgent need for simple *C. auris* tests, especially for surveillance samples. Lateral flow assays (LFAs), also known as lateral flow immunoassay or immunochromatographic assay, could be a good choice as they are rapid, cheap, stable, and easy-to-implement for presumptive identification of microbes (Koczula and Gallotta, 2016; Boutal et al., 2018). At the other end of the service spectrum, many hospital laboratories already use multiplex test panels for *Clostridioides difficile* and carbapenem-resistant Enterobacterales (CRE), and synergy of such panels with *C. auris* testing would be welcome (Binnicker, 2015; Crobach et al., 2016; Kost et al., 2017; McDonald et al., 2018; Bogaerts et al., 2020; CDC, 2021c). Current AFST services are inadequate as turnaround time is woefully inadequate, and new test formats/devices are needed for faster reporting and AFST-directed treatment of *C. auris* infections. Laboratory surveillance is crucial for monitoring and control of *C. auris* outbreaks. *C. auris* genome analysis has a proven role in the control of hospital outbreaks by pinpointing the common source (Eyre et al., 2018; Theodoropoulos et al., 2020). However, fungal genome sequencing and analysis remain beyond the capabilities of most diagnostic laboratories. Therefore, local outbreak investigations require diagnostic laboratories to quickly access regional and national collaborative networks with standardized *C. auris* sequencing tools (Ladner et al., 2019; Mintzer et al., 2019; NIHR Global Health Research Unit on Genomic Surveillance of AMR, 2020).

## Author Contributions

ED: draft preparation, review and editing, visualization. SC: supervision, conceptualization, and review and editing. VC: supervision, conceptualization, draft preparation, and review and editing, visualization. All authors approved the final version.

## Conflict of Interest

The authors declare that the research was conducted in the absence of any commercial or financial relationships that could be construed as a potential conflict of interest.

## Publisher’s Note

All claims expressed in this article are solely those of the authors and do not necessarily represent those of their affiliated organizations, or those of the publisher, the editors and the reviewers. Any product that may be evaluated in this article, or claim that may be made by its manufacturer, is not guaranteed or endorsed by the publisher.
